# Effects of light colors on the biochemical composition of *Scenedesmus obliquus*

**DOI:** 10.1007/s42770-026-01957-1

**Published:** 2026-05-03

**Authors:** Vanessa Campos Guedes, Jaqueline Carmo da Silva, Antonio Carlos Luperni Horta, Ana Teresa Lombardi

**Affiliations:** 1 Departamento de Engenharia Química, Universidade Federal de São Carlos, São Carlos, SP Brasil; 2 Departamento de Botânica, Universidade Federal de São Carlos, São Carlos, SP Brasil

**Keywords:** *Scenedesmus obliquus*, Protein production, Carotenoid production, Microalgae metabolism light influence

## Abstract

This study explores the impact of different light colors on the population growth, photosynthesis, and biochemical composition of *Scenedesmus obliquus*. Literature shows various biotechnological solutions to mitigate the environmental problems caused by greenhouse gas emissions from environmentally unsustainable processes, and microalgae cultures have emerged as a promising technology with the potential to support diverse production chains. In this context, *Scenedesmus* has demonstrated considerable potential. The present study presents a straightforward approach to enhance protein production in *S. obliquus* through the strategic manipulation of light wavelengths, with the potential for practical implementation. The paper goes on to outline future research directions for integrating light control technologies, emphasizing the potential for maximizing biomolecules accumulation. Cell density and viability, growth rates, carbohydrates and proteins, total carotenoids and photosynthesis inferred by pulse amplitude modulated fluorescence (PhytoPAM) were evaluated. Blue light was identified as optimal for total proteins production (5.5 µg/mL vs. 2.5–3.8 µg/mL in other light colors), while white light showed promise for carotenoid accumulation (0.160 µg/mL vs. 0.10–0.13 in other light colors). Notably, the observed variations in the light spectrum did not result in any significant changes in the maximum growth rates (1.08–1.13), underscoring the robustness of the organism under study. Photosynthetic parameters remained unaffected by light quality, indicating a robust photosynthetic machinery (qP 1.0, NPQ 0.2, and effective quantum yield 0.58). These findings contribute to our understanding of algal biology and provide practical insights for biotechnological applications, paving the way for sustainable solutions.

**Supplementary Information:**

The online version contains supplementary material available at 10.1007/s42770-026-01957-1.

## Introduction

Microalgae are photosynthesizing organisms that play a fundamental role in natural aquatic ecosystems as primary producers and as a source of important biomolecules. These organisms are capable of synthesizing compounds of industrial interest, including but not limited to carbohydrates, proteins, lipids, and pigments such as carotenoids [1, 2]. Aiming at optimizing production the biochemical composition of microalgae can be manipulated in response to changing the environmental conditions. This possibility arises in reason to the microalgae physiological plasticity, whereby increased biomolecules are produced as an adaptation response [3]. Among the biochemical manipulation techniques, changes in nutrient composition and light colors have been proposed [4]. As photoautotrophic organisms, microalgae depend on light to carry out photosynthesis, which is a process of transforming light energy into biochemical energy used by the cell to grow and survive [5]. The light energy offered to microalgae has two important factors that act on the development of the microorganism, the intensity and wavelength.

The light intensity is defined as the photon flux in a given area over a specified time interval. This variable can limit microalgal growth if its value is not sufficient, as well as photo inhibit this growth in case it is excessive [6]. However, the use of excessive or lower light intensity than the optimal can be important in the optimization processes of microalgal products. Unwanted light values ​​cause cellular stress and end up triggering microalgal defense mechanisms, which can increase the production of a cellular biochemical compound, or even modify its physiological parameters, changes that may be of interest to the biotechnology industry.

The production of a specific bioproduct from a microalgae/cyanobacteria species can be increased by considering both the intensity and the quality of the irradiated wavelength given to the culture composition. According to several studies, when modifying the wavelength during cultivation, the biochemical composition of the species also changes [7–10] and these changes are species-specific and related to the evolutionary history of the organism [11]. Investigating *Chlorella vulgaris* exposed to different light colors, Esteves et al., [8] obtained enhanced lipids under white light, while blue light increased the protein content, and red light carbohydrates. Chen et al., [7] studied *Spirulina platensis* in shaking flasks illuminated with blue light and observed it was best for specific pigments production (chlorophyll *a* and phycocyanin) at high light intensity, 3000 µmol photons m^− 2^ s^− 1^, but red light was better for increasing growth rate. Similarly, Shu et al., [10] cultivated *Chlorella* sp. in a bubble column reactor and showed that red light stimulated growth, while blue light was optimum for oil formation. Kim et al., [9] stated that more significant physiological changes were observed under red than blue light in cultures of *Nannochloropsis gaditana* and showed that red illumination was useful for lipid production.

Considering that different qualities of light can be used for biochemical manipulation of microalgae, targeting molecules of commercial interest, this approach becomes attractive for bioprocesses. Although several studies have evaluated the influence of light spectrum on microalgal growth, limited information is available regarding how specific monochromatic and combined light wavelengths affect the biochemical composition of *Scenedesmus obliquus* under controlled photoperiod conditions. Therefore, it is essential to investigate the species’ biochemical composition under defined spectral treatments in order to better understand metabolic allocation within the biomass. In this context the present experimental study primarily aimed to evaluate protein accumulation in *Scenedesmus obliquus* when subjected to blue, red, white, and a mixture of red and blue light conditions, while also assessing changes in carbohydrates, lipids, and pigments. This approach allows the identification of spectral conditions that may enhance the accumulation of target biomolecules for biotechnological applications.

## Materials and methods

### Culture conditions

**Fig. 1:**
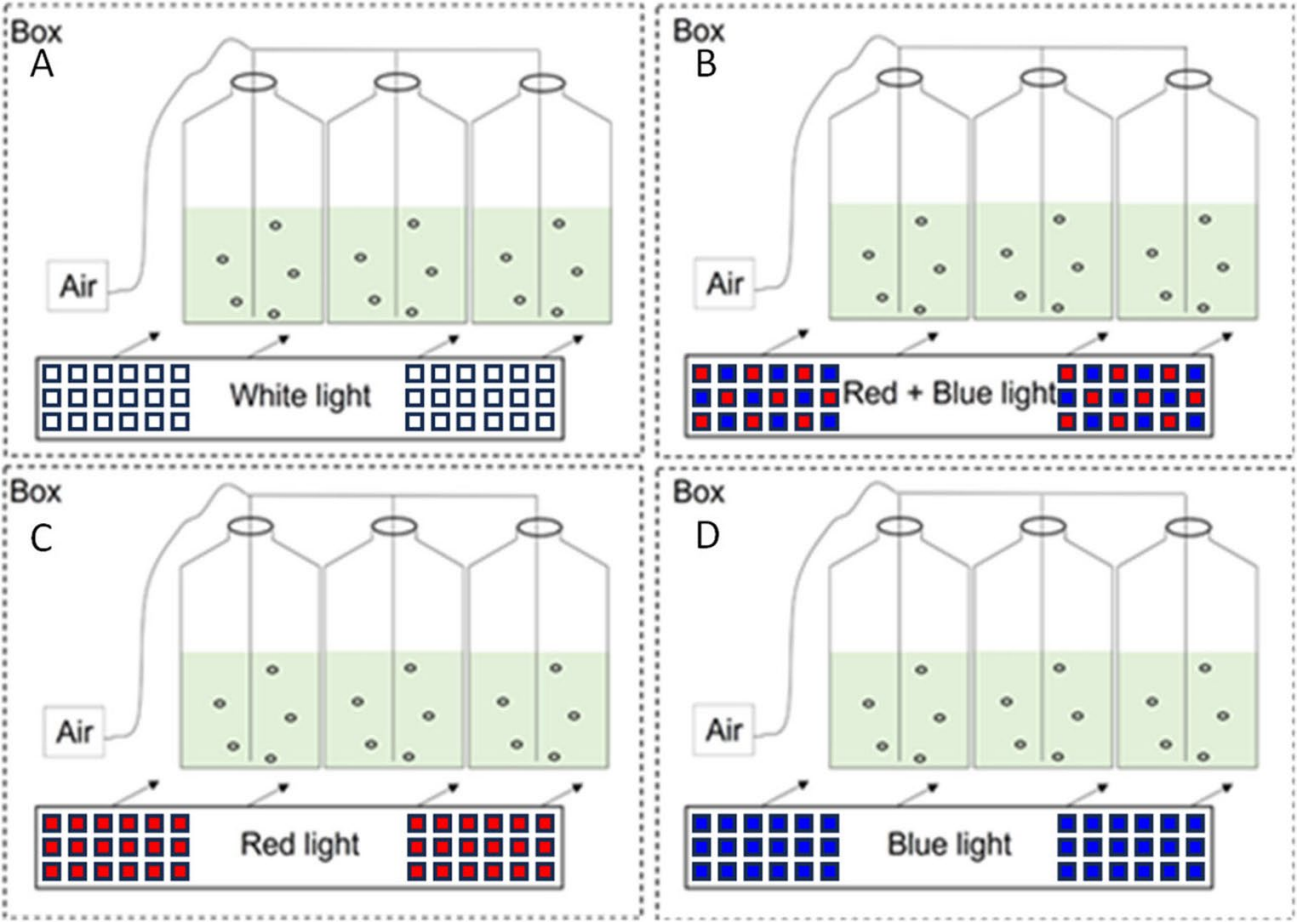
Experimental setup showing the four independent light treatments applied to *Scenedesmus obliquus* cultures: (**A**) white light, (**B**) combined red and blue light, (**C**) red light, and (**D**) blue light. Cultures were maintained in isolated opaque boxes with continuous aeration and identical temperature and irradiance conditions. The combined red–blue treatment consisted of LEDs arranged in equal proportion

The freshwater microalga *Scenedesmus obliquus* (CCMA 604) was obtained from the microalgae culture collection of the Botany Department at Federal University of São Carlos, Brazil). Monoalgal cultures were grown in 600 mL tissue culture flasks containing 600 mL sterile and modified BG-11 [12]. Culture medium sterilization was performed through autoclaving (121 °C, 20 min) in glass flasks, while the tissue culture flask was sterilized by ethanol 70% rinse. Exponentially growing cells were inoculated into the culture medium at an initial density of 10^5^ cell mL^− 1^, and the experiments were conducted for 120 h, corresponding to the end of the exponential growth phase under the tested conditions. The treatments consisted of exposure the cells to four independent light conditions: white light, combined red–blue light, red light, and blue light. All treatments were done with three experimental replicates and submitted to 12/12 h light/dark cycle, at 24 ± 1º C and the light intensity of 200 µmol photons m^− 2^ s^− 1^. The 12:12 photoperiod was selected to simulate environmentally relevant conditions and to evaluate metabolic responses under alternating light–dark phases, rather than maximizing biomass productivity. Air was introduced in the culture during the entire cultivation period without interruption by bubbling. The cultures were isolated from ambient light through an opaque box, as shown in Fig. 1. All culture manipulation (inoculation and sampling) was done in a laminar flow cabinet.

The experiments were monitored daily for population density, cell viability, chlorophyll *a in vivo* concentration and maximum photosynthetic quantum yield. On the third culture day (exponential phase), samples were collected for biochemical analysis: total carbohydrates and proteins, total carotenoids, and photosynthetic parameters.

### Growth parameters

Optical density (OD) was determined daily at a wavelength of 684 nm to measure microalgal growth (Leduy and Therien 1977), using a spectrophotometer (NANOCOLOR, Macherey-Nagel, Germany). The cells number per volume were quantified using a cell counting chamber (Fuchs-Rosenthal) under optical microscope (Nikon Eclipse model E200, Japan). Chlorophyll *a* was determined by “in vivo” fluorescence (Turner Designs, Trilogy, USA). For this, a calibration curve was constructed by plotting the concentration of chlorophyll *a* extracted from exponentially growing *Chlorella vulgaris* cells [13] against “in vivo” chlorophyll *a* fluorescence (Chl *a*; µg mL^− 1^). The linear section of this experimental plot was fitted using linear regression and used to calculate the concentration of chlorophyll *a*. Cell viability was analyzed using a cytometer Muse^®^ Cell Analyzer (Merck Millipore, USA) and it is expressed as percentage of viable cells in relation to the total number of cells analyzed by cytometry.

The maximum specific growth rate (µmax) was calculated by linear regression of the natural log of cell density *versus* time (days) and chlorophyll *a* concentration *versus* time (days) for the exponential growth phase.

### Photosynthetic analysis

The evaluation of photosynthetic parameters included measurements acquired using a pulse amplitude modulated fluorimeter (PHYTO-PAM, Heinz Walz GmbH, Germany) at 22 °C temperature, where the five fundamental levels of fluorescence were obtained from PAM fluorometry. Before measurements, all samples were dark adapted for 20 min. Low intensity modulated light (1 µmol photons m^− 2^ s^− 1^) was provided to measure the basal fluorescence (F_0_) [14]. A saturating pulse of 2000 µmol photons m^− 2^ s^− 1^ was applied during 0.2 s and maximum fluorescence of dark-adapted cells (F_M_) recorded. With these two parameters the maximum quantum yield (ф_M_), was calculated, according to Eq. 1.

1 $$\phi_M=\frac{F_M-F_O}{F_M}$$

Effective quantum yield was evaluated during the exponential growth phase. The actinic light was set at 130 µmol photons m^− 2^ s^− 1^ and light pulses (0.2 s duration) every 10 s were applied for 10 min and steady state fluorescence (F_S_) and maximum fluorescence in light adapted state were measured (F’_M_). From these parameters the effective quantum yield (ф’_M_) was calculated as in Eq. 2.

2 $$\phi^{\prime}_M=\frac{F^{\prime}_M-{F_S}}{F^{\prime}_M}$$

The minimum fluorescence of light-adapted cells (F’_0_) was calculated from Eq. 3, proposed by Oxborough and Baker [15].

3 $$\:{F^{\prime\:}}_{0}=\frac{{F}_{0}\:}{\frac{{F}_{V}}{{F}_{M}}+\:\frac{{F}_{0}}{{F{\prime\:}}_{M}}}$$

The estimated value of two types of quenching according to Juneau et al., [14], were calculated from the dark and light adaptation parameters. Photochemical quenching (qP), calculated by Eq. 4, indicates the fraction of light energy destined to photochemistry. The NPQ quenching, calculated by Eq. 5, describes the dissipation of energy, mostly by heat, a mechanism used as defense against excess light and oxidative damage.

4 $$\:qP=\:\frac{{F^{\prime\:}}_{M}-{F}_{S}}{{F^{\prime\:}}_{M}-\:{F^{\prime\:}}_{0}}$$

5 $$\:NPQ=\:\frac{{F}_{M}-{F^{\prime\:}}_{M}}{{F^{\prime\:}}_{M}}$$

### Biochemical analysis

The determination of total carbohydrates (Eq. 6) followed the method of Albalasmeh et al., [16]. A glucose standard solution (1 mg mL⁻¹) was used to construct the calibration curve (0–100 µg mL⁻¹), and absorbance was measured at 315 nm. The linear regression obtained (R² = 0.999) is presented in Supplementary Material Figure S1. For this, 10 mL of exponentially growing cultures were centrifuged (4400 rpm, 20 °C, 15 min) and the pellet kept at -22 °C until analysis.

For protein determination, 50 mL of exponentially growing culture were centrifuged (4400 rpm, 20 °C, 15 min) and the pellet was stored at -22 °C until analysis. Microalgae protein quantification was performed according to Bradford [17], while the extraction procedure followed Rausch [18]. Bovine serum albumin (BSA; 1 mg mL^− 1^) was used as a standard to construct the calibration curve (0–80 µg mL⁻¹), with OD measured at 595 nm. The parameters of the linear regression are described in Eq. 7 (R²=0.999), and the corresponding calibration curve is presented in Supplementary Figure S2.

6 $$\:\mathrm{C}\mathrm{a}\mathrm{r}\mathrm{b}\mathrm{o}\mathrm{h}\mathrm{y}\mathrm{d}\mathrm{r}\mathrm{a}\mathrm{t}\mathrm{e}\mathrm{s}\:\left({\upmu\:}\mathrm{g}\:{\mathrm{m}\mathrm{L}}^{-1}\right)=\:0.015\times\:{OD}_{315\:nm}-0.002$$

7 $$\:\mathrm{P}\mathrm{r}\mathrm{o}\mathrm{t}\mathrm{e}\mathrm{i}\mathrm{n}\:\left({\upmu\:}\mathrm{g}\:{\mathrm{m}\mathrm{L}}^{-1}\right)=\:0.0099\times\:{OD}_{595\:nm}-0.0125$$

Total carotenoids and chlorophylls were determined according to Wellburn [19] in 3 mL of exponentially growing culture. The total chlorophyll was determined as in Shoaf and Lium [13] using DMSO as extraction solvent, due to its high efficiency in solubilizing chlorophylls and carotenoids without the need for mechanical cell disruption. Pigment concentrations were calculated following the equations proposed by Jeffrey and Humphrey [20] using spectrophotometer readings 665, 649 and 480 nm.

### Statistical analysis

All comparisons made in this study were based on single-factor ANOVA (significance level 0.05; α = 5%), to indicate a statistically significant difference between the compared conditions of the four treatments. The comparisons that were indicated by ANOVA as statistically different were submitted to the Tukey’s range test with a confidence level of 95%. Statistical treatments were carried out using the software Microsoft Excel.

### Spectral composition

**Fig. 2:**
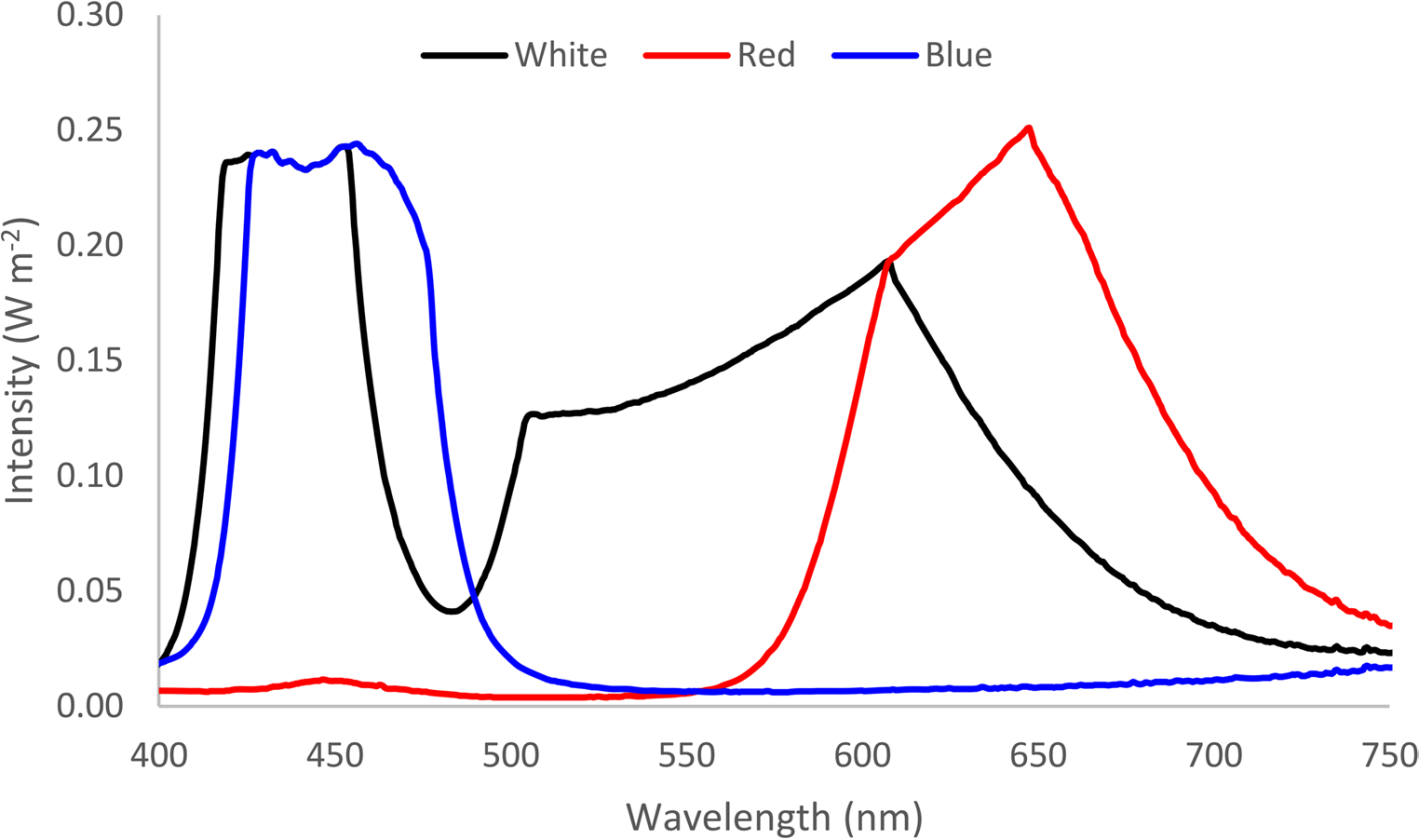
Emission spectrum of the four LED panels to which the cultures were exposed: white, blue, red and combination of blue and red

The LED (light-emitting diode) panels used had their spectral composition determined by a StellarNet Inc brand radiometer, model Fiber Optic Spectrometer. In the context of biochemical investigations, the analysis of light emitted by the panels is of fundamental importance, as it is through these data that accurate comparisons of microalgal behavior can be ensured. However, the emission spectrum is frequently not adequately described in the literature, thereby imposing difficulties for more precise comparisons between published results In Fig. 2, we show the emission spectrum measured using the StellarNet Inc radiometer, Fiber Optic Spectrometer model for the LED lights the cells were exposed.

## Results

### Growth parameters

**Fig. 3:**
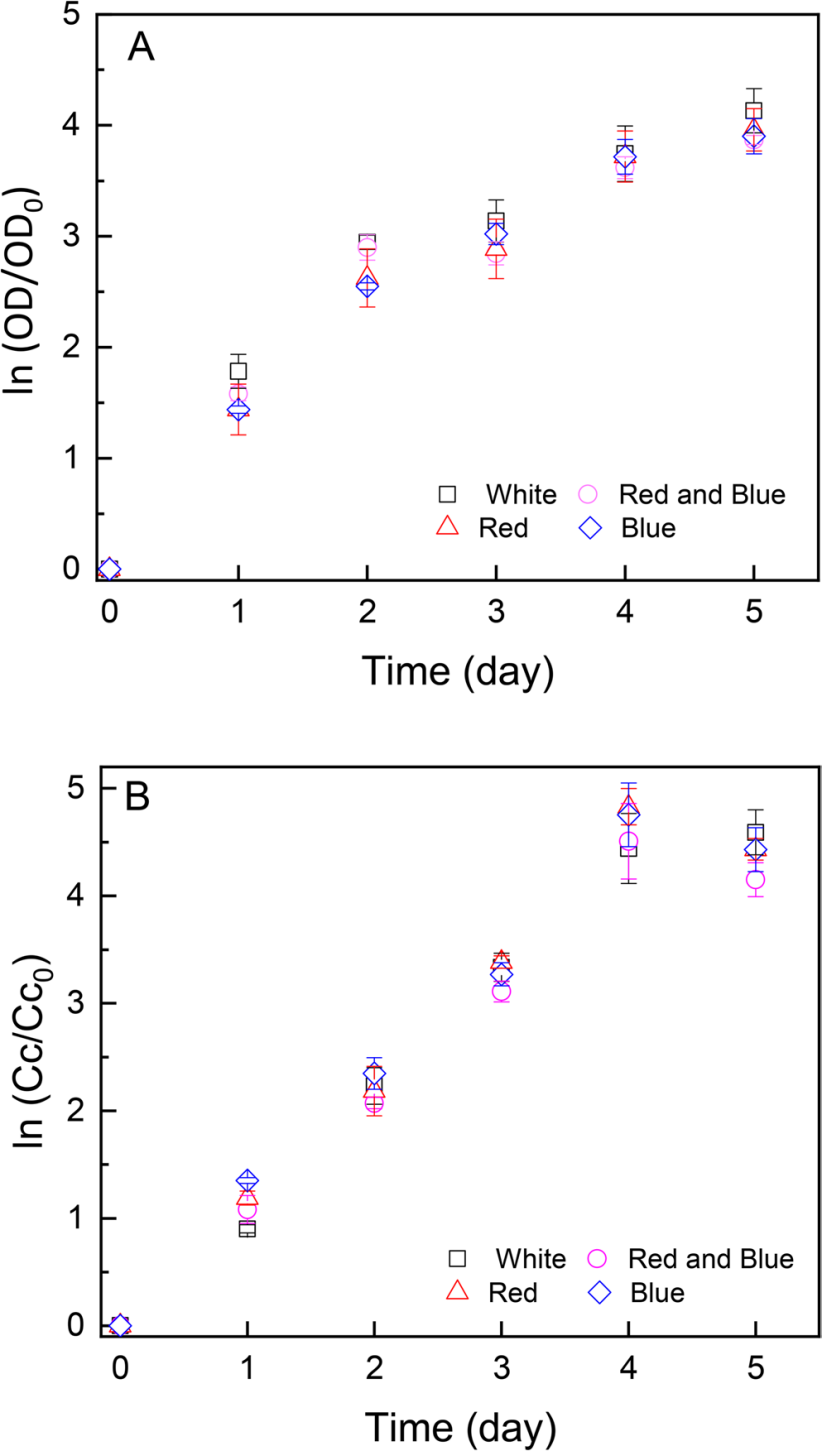
Growth curve for cultures of *Scenedesmus obliquus* grown under different light qualities (White, Blue, Red, and combination Red and Blue) as a function of optical density at 684 nm (A) and as a function of chlorophyll *a in vivo* (B)

The monitoring of the population growth of *S. obliquus* submitted to the different light colors is presented in Fig. 3A, and the concentration of chlorophyll *a in vivo* in Fig. 3B, for the cultivation period.

The maximum specific growth rate (µmax) of the different treatments were obtained by linearly adjusting the data during the exponential phase, configured from day 0 to day 2 (visually identified by the curves in Fig. 3), and are shown in Table 1, for adjustments using absorbance and chlorophyll *a* concentration data.

**Table 1:** Maximum specific growth rate obtained for *Scenedesmus obliquus* grown under different light quality regimes based in chlorophyll a concentration data

Light quality	µmax (day^− 1^)
White	1.13 (± 0.06)
Red & Blue	1.03 (± 0.01)
Red	1.11 (± 0.03)
Blue	1.08 (± 0.07)

**Fig. 4:**
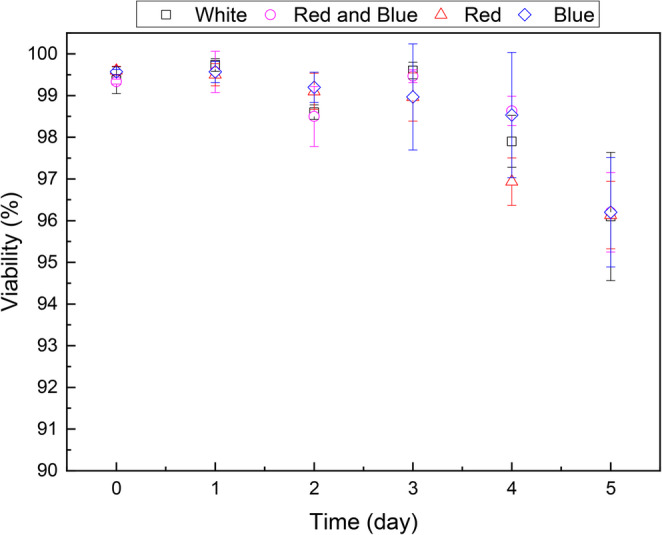
Cell viability (%) obtained from Muse Cell Analyzer for cultures of *Scenedesmus obliquus* kept under different LED light qualities as function of experimental time (days)

As demonstrated in Fig. 4, the wavelength of light exhibits minimal impact on cell viability data. The statistical analysis conducted reveals the absence of statistically significant differences between light qualities and cell viability of *Scenedemus obliquus* (*p* > 0.05).

### Photosynthetic parameters

**Fig. 5:**
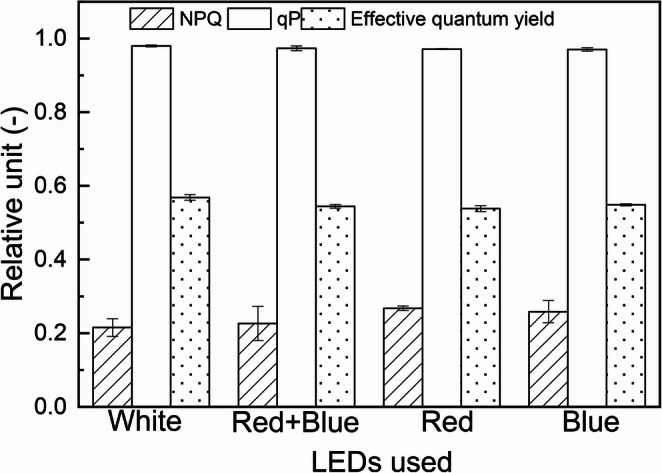
Photosynthetic data from cultures of *S. obliquus* illuminated with LED lights of different wavelengths (white, red & blue, red, blue). Photochemical quenching (qP), non-photochemical quenching (NPQ) and effective quantum yield

Figure 5 synthesizes the results of qP (photochemical), NPQ (non-photochemical), and the effective quantum yield. The statistical analyses indicated that there are no significant differences (*p* > 0.05) related to light wavelength/color and the photosynthetic parameters. The qP results suggest that light energy was directed toward photochemistry of photosynthesis in all light colors.

### Biochemical parameters

**Fig. 6:**
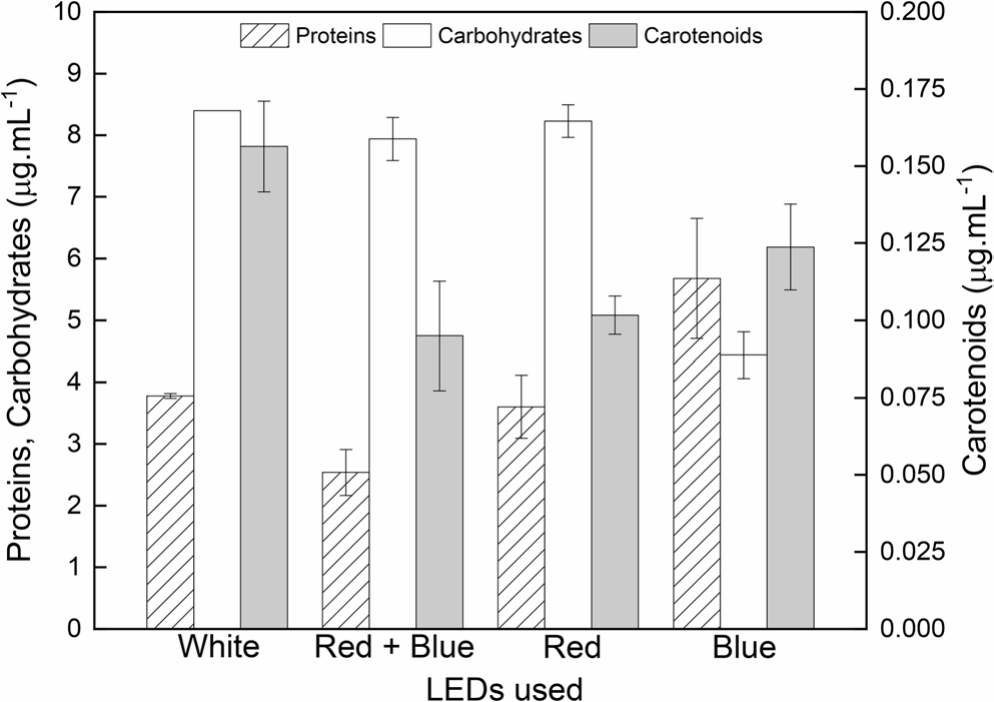
Biochemical composition results of *Scenedesmus obliquus* grown under the different LEDs colors: White, combined blue and red, Red, and blue. The left axis indicates the proteins and carbohydrates concentrations, while the right axis displays the carotenoids concentration

The results relative to the biochemical composition of the biomass obtained in cultures at the exponential phase, are shown in Fig. 6. It indicates that, despite the absence of discernible variations in photosynthetic parameters and growth rates across the different light colors, the synthesis of biomolecules was indeed influenced. Highest proteins were observed in *S. obliquus* exposed to blue LED (ANOVA *p* < 0.05).

In relation to carbohydrates, blue light again stood out, being the only one with significant differences in relation to other wavelengths, and responsible for the smallest amount of this compound (4.6 ± 0.4 µg mL^− 1^). It is important to note that all other comparisons had no statistical difference. Table 2 shows the ratio calculated with total carbohydrates/proteins accumulated by the microalgae at each wavelength.

**Table 2:** Carbohydrates/Proteins ratio for *Scenedesmus obliquus* grown under different wavelengths

Light color (LEDs used)	Carbohydrates/ Proteins
White	2.2 (± 0.1)
Red and Blue	3.2 (± 0.6)
Red	2.3 (± 0.4)
Blue	0.8 (± 0.2)

## Discussion

### Growth rate

Recent literature show that changes in light wavelength affect the physiological responses of microalgae by altering photosynthesis and the antenna pigments [21], as well as the biochemical composition of the cells [3, 8, 21], but not necessarily affect growth rate [3]. Our results showed that light colors, either red or blue, in comparison to white light, did not affect the growth rate of *S. obliquus*, in agreement to those in Aoyagi et al., [3]. The authors showed lack of statistically significant difference for *Kirchneriella contorta* growth rates obtained in cultures exposed to blue or red lights. Different from Aoyagi et al., [3] and the present results, Esteves et al., [8] obtained higher growth rate under red light in comparison to blue, orange and white lights for cultures of *Chlorella vulgaris*. However, Zhao et al., [21] showed that *Dunaliella salina* presented statistically similar growth rates under blue, red and white lights, but lower value under green light. Factors such as optical path and culture density, in addition to the energy per photon that is different for the different colors, can contribute to the differences observed. Zhao et al., [21] used low light intensity (40 µmol photons m^-2^ s^-1^) to grow *Dunaliella salina* and, therefore, light filtration and penetration in the culture medium were more protruding than in higher intensities. They showed that as time passed and culture density increased, just blue light stimulated the chlorophyll a and b. It can be concluded that in addition to the culture conditions, the effect of light quality on the growth of microalgae is species specific due to differences in metabolic pathways, pigmentation and photoreceptors [11].

Our results indicate that the cells were healthy regardless of the quality of light used, corroborating the observation that the emitted wavelengths did not impact the photosystem in *Scenedesmus obliquus*. However, it should be noted that this may not be the mandatory behavior for other species.

### Photosynthetic parameters

The photosynthetic responses of microalgae reflect the growing conditions. The maximum quantum yield of PSII ($\:{{\phi}}_{M}$) did not change with light quality (wavelength) in this study. We can observe that our experiments presented a stable profile between 0.74 and 0.76 throughout all cultivations, which Lombardi and Maldonado [22] classified as healthy microalgae values (0.6 to 0.8). In the present research, the health status of the cells is corroborated by more than 99% cell viability up to the third experimental day, in which the cultures were still exponentially growing.

The present results are in accordance with those presented in Baba et al., [23], who studied *Botryococcus braunii* and found no changes in $\:{{\phi}}_{M}$ in relation to red, green and blue light. Kamalanathan et al., [24] evaluated photosynthetic parameters of *Scenedesmus* sp. as a function of its growth under photoautotrophic, myxotrophic and heterotrophic conditions, and obtained $\:{{\phi}}_{M}$ values ​​for photoautotrophically cultivated microalgae between 0.68 and 0.73, approximately the same range found in this work. However, Zhao et al., [21] obtained different results. They showed that different light colors impacted on the photosynthetic quantum yield, with *Dunaliella salina* exposed to blue light having the highest values. This can be due to the effect of low light used by the authors (40 µmol photons m^− 2^ s^− 1^), whose penetration in the culture and selfshading may have been significant. Mattos et al., [25] showed that high density cultures of *Scenedesmus bijugus* absorbed better the lights (green) that penetrated deeper in the cultures.

The $\:{{\phi}^{\prime\:}}_{M}$ results we obtained suggest that the physiological state of the photosynthetic apparatus was healthy [22, 26], and that it was not altered by any of the wavelengths in which *Scenedesmus obliquus* was grown. It should be noted that this behavior may be different for other species, as in the case of diatoms that showed a reduction in $\:{{\phi}^{\prime\:}}_{M}$ in red light [27].

Confirming the absence of the light colors on photosynthesis in the present research, light energy was being directed to photochemistry of photosynthesis in all light wavelength tested, that is, PSII was converting the absorbed energy into biochemical energy [26], not being harmed or degraded as a result of the entropy. This is in agreement with the behavior obtained for *Botryococcus braunii*, where qP was not affected by the quality of light used [23]. One of the directions that energy not sent to the photochemical pathway can take is heat dissipation, which can be represented by NPQ [28]. According to literature [29] NPQ can be a response to stress caused by changes in nutrient concentrations or light excess, a via of self-protection by preventing excessive flow of electrons to PSI, thus reducing the potential of photoinhibition [30]. The present results showed no variation of NPQ as a function of light quality in *S. obliquus*, which agrees with the results found for *Botryococcus braunii* by Baba et al., [23]. As the cultures in this research were set to have the same PAR intensity, regardless of the color of light used, they were not exposed to excessive light. Given that the NPQ triggering mechanisms are determined by the evolutionary characteristics of the microalgae species [29], it is important to note that different species of microalgae can exhibit divergent responses to light in comparison to *Scenedesmus obliquus* in the present study.

Similar to qP and NPQ, no difference was observed for effective quantum yield of PSII ($\:{{\phi}^{\prime\:}}_{M})$. Thus, the present study highlights that the ability to move electrons beyond the PSII in the transport chain in a certain state of adaptation to irradiance, determined by the effective quantum yield coefficient [31], does not undergo any modifications in *S. obliquus* in regarding the lights wavelengths. Similar behavior was reported by Baba et al., [23] which indicated null effects on the $\:{{\phi}^{\prime\:}}_{M}\:$as a function of light colors (red, green and blue) in *Botryococcus braunii*. Mercado et al., [32] when studying five species of diatoms reported that changes in $\:{{\phi}^{\prime\:}}_{M}$ were not present in three of them, however two species presented variation in this photosynthetic parameter. They showed that *Nitzschia laevis* had higher $\:{{\phi}^{\prime\:}}_{M}$, whereas *Navicula uncertain* 45% lower under blue light compared to white light. Therefore, the responses of the photosynthetic apparatus to different light colors can, as growth rates, be related to the evolutionary history of the organism.

### Biochemical parameters

The regulatory role of spectral quality in modulating biomass composition and high-value metabolite accumulation has been extensively discussed in recent literature, emphasizing that red and blue wavelengths differentially affect photosynthetic efficiency and metabolic pathways [33, 34]. Blue light is situated in the lower wavelength range of the visible spectrum and caries approximately 57% more energy than red light, located at the opposite end of the spectrum. This higher photon energy influences light absorption dynamics within microalgal cultures [35]. Therefore, one potential mechanism by which blue light affects photosynthesis involves the surplus energy reaching the photosynthetic apparatus. This excess energy is often associated with increases in NPQ and pigment accumulation [29] however, in the present study, this photosynthetic parameter was not affected by blue light.

Another important influence of blue light has been reported in Ruyters [36] and is related to photosynthetic enzymes including those of pigment synthesis and photorespiration, and carbohydrate degrading enzymes. The author reported that these enzymatic alterations are commanded by two types of defense mechanisms, called coarse control and fine control. In coarse control, blue light can act on the amount of active enzyme and on the enzyme’s capacity, by changing the rate of synthesis or degradation. In the fine control, changes occur through regulation of the activity of preexisting enzymatic molecules, and an important indicator of this type of influence is the breakdown of carbohydrates. The defense mechanisms represented by the coarse and fine controls in *S. obliquus* may have been affected by blue light, since an increase in proteins with a concomitant decrease in carbohydrates were obtained, indicating that the influence may be related to the enzymes.

The lower value of carbohydrates can also be related to increase in O_2_ consumption under blue light as explained in Emerson and Lewis [37]. In their study, the authors observed a higher consumption of O_2_ in the dark phase after using blue light in the photosynthetic period compared to red light. Kowallik [38], explained that the increase in O_2_ uptake does not result from wavelength inhibition, but from interference by extra oxygen consumption, and that additional carbohydrate degradation occurs even during photosynthesis, independent of the photosynthetic machinery, when the organism is exposed to blue light.

Schulze et al., [39] obtained a stimulation by blue light under the use of nutrients such as N-NO_3_ in *Tetraselmis chuii*, when compared to red light, also indicating a negative effect on carbohydrate-protein ratios at wavelengths of 400–450 nm. Kim et al., [9] found 50% greater use of phosphorus when *Scenedesmus* sp. was exposed to blue light, in comparison to white light. Information that fully agrees with the results presented in Table 2 in this study, which shows a significant difference for all wavelengths in relation to blue. However, this increase in nutrient use is linked to the activation of transporters/enzymes responsible for NO_3_ and P assimilation. An indicator of this phenomenon is high C: N ratios and higher levels of carbohydrates or lipids rather than proteins [40]. This possibility was then discarded in the present study, because the blue light was responsible for lower carbohydrates and higher proteins, counteracting the inhibition indicator due to lack of nutrients. The absence of nutrient limitation in this case is also confirmed by the maximum growth rate values, similar for all treatments.

Regarding the analysis of carotenoids, statistically significant differences were indicated between white light and the mixture (blue and red), and between white light and red light. All other comparisons indicated that there was no statistically significant difference. The average total carotenoids production in white light condition (0.156 µg mL^− 1^) was higher than that of red light (0.102 µg mL^− 1^) and mixed light (0.095 µg mL^− 1^). As is well known, carotenoids fulfill two primary functions within the chloroplast. They serve as photosynthetic pigments and photoprotective agents, offering resilience against adverse environmental conditions involving excess light [41]. Similar wavelength-dependent responses have been reported for *Chlorella sorokiniana*, in which red LED illumination enhanced pigment accumulation, while additional abiotic stress favored lipid production under a two-stage cultivation strategy [34]. These findings reinforce that spectral quality can modulate pigment biosynthesis, although the magnitude and direction of the response may vary depending on species and cultivation conditions.

The difference between white light in relation to the mixture (red and blue) and red light can be explained according to the composition of the spectrum of the used LEDs (Fig. 2) and by the pigments of the chlorophytes. Therefore, the explanation is based on the widely accepted information that red and blue light are intensely absorbed by chlorophyll *a* (Madigan et al. 2016). Consequently, the comparison of white light in this study, which is composed of a more complete wavelength range also including blue and red, indicated that the microorganism evolved carotenoids capable of utilizing these energies outside the optimal absorption peaks. This finding explains the greater production in white light compared to mixed light and red light.

## Conclusion

In conclusion, our study demonstrates the remarkable consistency of maximum growth rates of *Scenedesmus obliquus* across different light wavelength, underscoring the resilience and adaptability of the microorganism. Despite variations in light spectra, photosynthetic parameters remained unaffected, indicating the robustness of its photosynthetic machinery. The findings of this study further suggest that the optimization of protein production in *S. obliquus* cultures can be attained through the implementation of a pragmatic and efficient strategy for the manipulation of light wavelengths.

Moreover, our research reveals a promising avenue for future exploration in light control technology, where the integration of light intensity and wavelength manipulation holds the key to maximizing productivity. Specifically, blue light emerges as the optimal wavelength for maximizing total protein production, while other light qualities may be more appropriate for specific biochemical objectives such as carbohydrate synthesis. In addition, our results highlight the significant advantages of using white light for carotenoid production, which offers considerable improvements over narrower wavelength range light sources.

In essence, our study not only contributes to the understanding of *Scenedesmus obliquus* biology and photosynthesis but also provides practical insights for biotechnological applications. The strategic exploitation of light manipulation has emerged as a promising approach, offering novel prospects for enhancing the productivity and adaptability of microalgae-based systems. This advancement signifies a substantial step forward in the direction of sustainable solutions for environmentally responsible industrial bioprocess.

## Supplementary Information

Below is the link to the electronic supplementary material.

Supplementary Material 1

## Data Availability

All data that support the findings of this study are available in the paper. Raw data can be accessed upon request to the authors.
