# Supplementary material for: Effects of light colors on the biochemical composition of Scenedesmus obliquus
Source: Braz J Microbiol. 2026 May 3;57(1):132. doi: 10.1007/s42770-026-01957-1 (PMC13136458; doi:10.1007/s42770-026-01957-1)
Supplement: Supplementary file 1 — Supplementary Material 1 [file 42770_2026_1957_MOESM1_ESM.docx]

Figure S1. Calibration curve for total carbohydrate quantification using glucose as standard (0–100 µg mL⁻¹), according to Albalasmeh et al. (2013). Optical density was measured at 315 nm. The linear regression equation was Glucose (µg mL⁻¹) = 0.015 × OD₃₁₅ − 0.002, with a correlation coefficient (R²) of 0.999. Error bars represent standard deviation of triplicate measurements.

Figure S2. Calibration curve for total protein quantification using bovine serum albumin (BSA) as standard (0–80 µg mL⁻¹), according to Bradford (1976). Optical density was measured at 595 nm. The linear regression equation is described in Equation 14, with a correlation coefficient (R²) of 0.999. Error bars represent standard deviation of triplicate measurements.
